# Distribution of Constituents and Metabolites of Maritime Pine Bark Extract (Pycnogenol^®^) into Serum, Blood Cells, and Synovial Fluid of Patients with Severe Osteoarthritis: A Randomized Controlled Trial

**DOI:** 10.3390/nu9050443

**Published:** 2017-04-28

**Authors:** Melanie Mülek, Lothar Seefried, Franca Genest, Petra Högger

**Affiliations:** 1 Institut für Pharmazie und Lebensmittelchemie, Universität Würzburg, 97074 Würzburg, Germany; melanie.muelek@pharmazie.uni-wuerzburg.de; 2 Department of Orthopedics, Orthopedic Center for Musculoskeletal Research, 97074 Würzburg, Germany; l-seefried.klh@uni-wuerzburg.de (L.S.); f-genest.klh@uni-wuerzburg.de (F.G.)

**Keywords:** pine bark extract, LC-ESI/MS/MS, randomized controlled study, osteoarthritis, human, polyphenols

## Abstract

The present randomized controlled study aimed to investigate the in vivo distribution of constituents or metabolites of the standardized maritime pine bark extract Pycnogenol^®^. Thirty-three patients with severe osteoarthritis scheduled for a knee arthroplasty were randomized to receive either 200 mg per day Pycnogenol^®^ (P+) or no treatment (Co) over three weeks before surgery. Serum, blood cells, and synovial fluid samples were analyzed using liquid chromatography coupled to tandem mass spectrometry with electrospray ionization (LC-ESI/MS/MS). Considerable interindividual differences were observed indicating pronounced variability of the polyphenol pharmacokinetics. Notably, the highest polyphenol concentrations were not detected in serum. Catechin and taxifolin primarily resided within the blood cells while the microbial catechin metabolite δ-(3,4-dihydroxy-phenyl)-γ-valerolactone, ferulic, and caffeic acid were mainly present in synovial fluid samples. Taxifolin was detected in serum and synovial fluid exclusively in the P+ group. Likewise, no ferulic acid was found in serum samples of the Co group. Calculating ratios of analyte distribution in individual patients revealed a simultaneous presence of some polyphenols in serum, blood cells, and/or synovial fluid only in the P+ group. This is the first evidence that polyphenols distribute into the synovial fluid of patients with osteoarthritis which supports rationalizing the results of clinical efficacy studies.

## 1. Introduction

Dietary polyphenols have been associated with numerous beneficial effects on human health. Studies investigating the absorption of polyphenols from the gastrointestinal tract revealed that blood concentrations of individual polyphenols are often very low [1]. Moreover, polyphenolic compounds are often subjected to an extensive metabolism [2]. Some metabolites generated by gut microbial metabolism obviously contribute to health effects [3].

One of those bioactive metabolites is δ-(3,4-dihydroxy-phenyl)-γ-valerolactone (M1) which is formed by the human intestinal flora from the procyanidins’ catechin units [2]. It has been detected in urine and plasma samples after intake of Pycnogenol^®^ [4,5]. The dietary supplement Pycnogenol^®^ is a standardized extract of the French maritime pine, which conforms to the monograph “*Maritime pine extract*” in the United States Pharmacopeia (USP). It contains 65%–75% oligomeric procyanidins and polyphenolic monomers, phenolic, or cinnamic acids and their glycosides [6]. In numerous clinical studies Pycnogenol^®^ demonstrated effects in different chronic diseases of, for example, inflammatory or cardiovascular origin [6,7].

Another chronic disease with high pharmacoeconomic burden and significant impact on the patients’ quality of life is osteoarthritis (OA). OA is a chronic degenerative joint disease which is characterized by progressive cartilage destruction and it is the leading cause of pain and disability [8]. Treatment of OA includes pharmacological and non-pharmacological interventions and aims at pain relief and improvement of function. Severe OA might also require surgical interventions such as knee or hip arthroplasty [9]. Dietary factors or supplements have been discussed as options in the management or prevention of OA [10].

In clinical studies OA symptoms such as pain and joint stiffness have been shown to improve upon intake of Pycnogenol^®^ [11,12]. While this clinical observation is consistent with a previously shown inhibition of nuclear factor κB (NF-κB) activation and inhibition of various matrix metalloproteinases by constituents or metabolites of this pine bark extract [13,14], it is not clear yet whether bioactive polyphenols would actually be present at the site of disease (e.g., in the joints affected by OA). After an oral intake of multiple doses of Pycnogenol^®^ concentrations in the nanomolar range of catechin, taxifolin, caffeic acid, ferulic acid, and of a bioactive metabolite M1 have been detected in human plasma [5]. Moreover, an uptake of M1 into erythrocytes, monocytes, and endothelial cells has been observed in vitro [15,16]. The purpose of the current study was to investigate the in vivo distribution of constituents or metabolites of Pycnogenol^®^ in serum, blood cells, and synovial fluid of patients with severe OA scheduled for a knee replacement surgery.

## 2. Materials and Methods

### 2.1. Clinical Study Design

The present study was a randomized controlled clinical trial involving patients with severe osteoarthritis (OA) according to the Western Ontario and McMaster Universities Arthritis Index (WOMAC) score, who were scheduled for an elective knee replacement surgery (Kellgren-Lawrence grade III–IV). Patients were not eligible if they regularly took non-steroidal anti-inflammatory drugs (NSAIDs) or glucocorticoids perorally within the past four weeks, if they currently received a therapy with anti-coagulants, or if they tested positive for the human immunodeficiency virus (HIV), hepatitis B or C, or if they had a previous or current infection of the affected knee joint. As rescue medication acetaminophen (paracetamol), tramadol, or a combination of tilidine and naloxone was allowed. The study was conducted in accordance with the Declaration of Helsinki, and the protocol was approved by the local Ethics Committee of the Medical Faculty of the University Würzburg (Project identification code 248/11).

A total of 33 OA patients were recruited for the study and gave informed written consent before they participated in the study. The chosen number of patients was based on a previous pharmacokinetic study with healthy volunteers receiving the pine bark extract [5]. Patients were randomized into two groups using a computer-generated randomization list which was not accessible to the physicians and nurses who were involved in the patient care and management. Study participants (*n* = 16) were assigned to the treatment group receiving 200 mg of the French maritime pine bark extract Pycnogenol^®^ (Horphag Research Ltd., Geneva, Switzerland) per day (twice daily two capsules with each 50 mg) over three weeks prior to the planned surgery. The control group was comprised of 17 patients who did not receive Pycnogenol^®^. All patients were asked to comply with a polyphenol-free nutrition, especially two days before each blood sampling. For this purpose, they were provided with nutritional check-lists specifying food/beverages they should avoid and for recording what they ingested within the last two days before blood sampling. Adherence to the study medication was estimated based on the number of returned Pycnogenol^®^ capsules upon hospitalization for the knee replacement surgery.

Blood samples from each study participant were collected (BD Vacutainer^®^ SST II Advance; Becton Dickinson GmbH, Heidelberg, Germany) before oral intake of Pycnogenol^®^ (V1, basal value); during the intake, approximately 1–2 days before the surgery (V2); and during or shortly before knee surgery (V3), about 12 h after the last dose of Pycnogenol^®^. Immediately after blood sampling the serum and cellular fraction were separated under sterile conditions. On the day of the surgery residual knee cartilage and synovial fluid were also collected. All samples were shock-frozen immediately and stored at −80 °C. The outcome measure was the concentration of pine bark extract-derived polyphenols in serum, blood cells, and synovial fluid as determined by liquid chromatography coupled to tandem mass spectrometry with electrospray ionization (LC-ESI/MS/MS).

All medical procedures including enrollment of participants, surgery, patient care, and sample collection took place at the orthopedic center (Orthopädie und Orthopädische Klinik König-Ludwig-Haus, Universität Würzburg) between September 2012 and September 2014. The generation of the random allocation sequence, assignment of participants to the intervention or control group, and analysis of all patient samples took place at the Institut für Pharmazie und Lebensmittelchemie. Since the study primarily focused on pharmacokinetic/bioanalytical aspects, specifically on the analysis of polyphenols in various human specimen, an early registration was overlooked and the study was registered retroactively.

### 2.2. Chemicals, Reagents, and Special Materials

Analytical standards of (+)-catechin, taxifolin, ferulic acid, caffeic acid, and the internal standard (IS) 3,4-dihydroxyhydrocinnamic acid (hydrocaffeic acid) were all obtained from Sigma-Aldrich (Taufkirchen, Germany). The metabolite M1 (δ-(3.4-dihydroxy-phenyl)-γ-valerolactone) was synthesized by M. Rappold as part of his diploma thesis. Methanol (MeOH, LC-MS analyzed) from J.T.Baker Mallinckrodt and water (HiPerSolv CHROMANORM^®^ for LC-MS) were obtained from VWR (Darmstadt, Germany). Ammonium formate (AF) and formic acid (FA) were purchased from Sigma-Aldrich. An enzymatic mixture of β-glucuronidase/sulfatase (β-Gln/Sulfa) from *Helix pomatia* (Type HP-2; Sigma-Aldrich) was used for enzymatic hydrolysis. Ethyl acetate, *tert*-butyl methyl ether (MTBE), and phosphate buffered saline (PBS, pH 7.4) were obtained from Sigma-Aldrich.

### 2.3. Standard Solutions

Stock solutions (1 mg/mL) of each standard substance ((+)-catechin, taxifolin, ferulic acid, caffeic acid, and M1) and of the internal standard (IS; hydrocaffeic acid) were prepared in 100% methanol and stored at −80 °C. They were diluted with methanol to yield working standards which were aliquoted and stored at −20 °C.

### 2.4. Human Specimen for Calibration Curves

Packed cells and serum were obtained from a blood bank (Bayerisches Rotes Kreuz (BRK), München, Germany) and handled as previously described [17,18]. Synovial fluid was collected from patients with intra-articular fluid accumulation who needed punctuation of the effusion for medical reasons. Synovial fluid samples were pooled to obtain a single batch for preparation of calibration standards for quantification of the clinical study samples.

### 2.5. Liquid Chromatography (LC)

Details of the LC method have been reported before [18,19]. Briefly, for the LC analysis an Agilent 1260 system was used. The chromatographic separation was carried out using a Pursuit PFP-C18 column (4.6 × 150 mm, particle size 3 µm) at 20 °C (all from Agilent Technologies, Santa Clara, CA, USA). The mobile phase consisted of 5 mM ammonium formate with 0.065% (*v/v*) formic acid (pH = 3.2; A) and methanol with 0.1% formic acid (B). The flow rate was set to 0.6 mL/min and the sample injection volume was 5 µL. The gradient elution was conducted starting at 60% B (0 min) to 95% B (2.50 min) and maintained to 95% B to 5.50 min followed by re-equilibration at 60% B. The total run time was 10.00 min with a post time of 3 min.

### 2.6. Mass Spectrometry (MS/MS)

Details of the MS/MS method using a G 6460 TripleQuad LC/MS with turbo electrospray ionization (ESI; Agilent Technologies, Santa Clara, CA, USA) have been previously reported [17,18]. The optimized MS/MS transitions and mass spectrometric parameters of the compounds to be quantified in human blood cell and serum samples were recently reported [18,19]; optimized parameters of additionally determined M1 metabolites in blood cells are listed in Table S1 in the electronic supplementary material. Optimized MS/MS transitions and mass spectrometric parameters of the compounds to be quantified in human synovial fluid samples are listed in Table S2.

### 2.7. Preparation of Human Serum Samples

Serum samples (1.5 mL) were prepared by liquid-liquid extraction with prior enzymatic incubation containing β-Gln/Sulfa to hydrolyze conjugated analytes [5], as previously described [18,19]. Additionally, 1.5 mL of serum was analyzed without prior enzymatic hydrolysis to calculate the degree of conjugation with sulfate and glucuronic acid.

### 2.8. Preparation of Human Blood Cell Samples

Human blood cell samples were prepared as previously detailed [18]. Therefore, 2.0 mL blood cells of each study volunteer were processed with prior enzymatic hydrolysis to determine the total concentration of the analytes.

### 2.9. Preparation of Human Synovial Fluid Samples

Synovial fluid samples were prepared with a newly developed and optimized liquid-liquid extraction method. Therefore, 40 µL 4% o-phosphoric acid was added to 1.0 mL human synovial fluid (pH 5.0). Afterwards, the samples were incubated with an enzyme mixture containing β-Gln/Sulfa (1500 U β-Gln and 2 U Sulfatase per mL synovial fluid) for 45 min at 37 °C on a horizontal shaker (100 rpm) to hydrolyze conjugated analytes [5]. Then, 60 µL 4% o-phosphoric acid (pH 3.2), 25 µL IS (24.85 ng/mL), and 3.0 mL extraction solvent containing ethyl acetate and *tert*-butyl methyl ether (1:1; *v/v*) were added, vortexed for 1 min (Multi-Vortex, VWR, Darmstadt, Germany), and centrifuged for 5 min at 3300× *g* (4 °C). Thereafter, 2.0 mL of the upper organic layer was evaporated to dryness under nitrogen. The residue was reconstituted in 75 µL of 100% MeOH and centrifuged at 18,000× *g* for 15 min at 4 °C before LC-MS/MS analysis.

A full validation was performed for the quantification of the analytes in human synovial fluid with the optimized liquid-liquid extraction method and prior enzymatic hydrolysis. The validation included the selectivity, linearity, lower limit of quantification (LLOQ), accuracy and precision (intra- and interday), recovery, process efficiency, matrix effects (quantitative), carry over, cross talk, and post-preparative stability. Also, the freeze- and thaw-, short-term-, and long-term stability of the analytes in human serum were investigated.

### 2.10. Quantification of the Samples of the Study Participants

For each patient, specimen human pooled matrix-matched calibration standards with an internal standard (structural) were used for quantification of the study samples. In case of a basal presence of an analyte in the blank matrix, the calibration curve was shifted along the y axis by the response of the zero-sample (containing the IS) [20].

### 2.11. Statistical Analysis

Data were analyzed using descriptive statistics. Typically, mean and standard deviation (SD) were calculated. For comparison of the study participants’ basic demographic characteristics a Student’s *t*-test was used to compare the patients of the treatment and control group.

## 3. Results

### 3.1. Patients and Protocol Adherence

**Figure 1:**
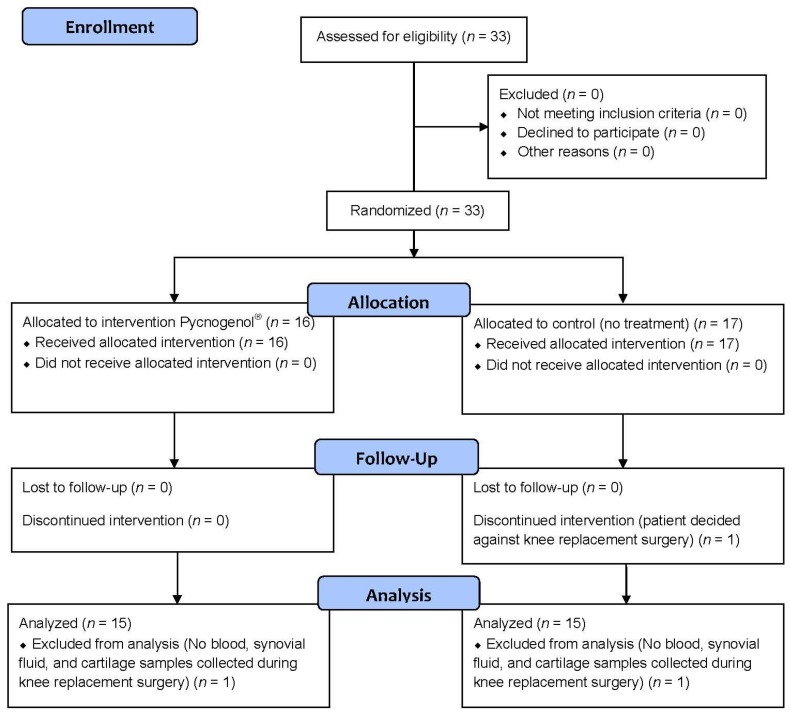
CONSORT (Consolidated Standards of Reporting Trials; www.consort-statement.org) 2010 Flow diagram of the study.

A total of 33 patients were enrolled into the study and randomized to receive either Pycnogenol^®^ (*n* = 16) or no treatment (*n* = 17). One patient of the control group decided against the scheduled knee replacement surgery and was excluded. During the surgical procedure, there was a failure to collect blood, synovial fluid, and cartilage samples for one patient in both the control group and the Pycnogenol^®^ group. These patients were excluded from the analysis (Figure 1). Thus, 30 patients (66.7% female) underwent analysis after receiving Pycnogenol^®^ (“P+”; 9 females, 6 males) or no treatment as the control group (“Co”; 11 females, 4 males). There was no statistically significant difference between the groups in any of the basic demographic characteristics (Student’s *t*-test, *p* > 0.05), the mean age (± standard deviation SD) was 64.3 ± 8.2 years, height 1.69 ± 0.10 m, body weight 87.33 ± 15.66 kg (BMI 30.74 ± 5.29 kg/m^2^).

All study participants were requested to avoid polyphenol-rich food/beverages (e.g., coffee, green tea, wine, chocolate, some fruits and vegetables) within the last two days before the blood samplings. Analysis of the nutrition protocols revealed that the nutritional advice was not followed well and dietary violations were admitted before collecting 42% of the blood samples. Thus, concentrations of common polyphenols such as catechin or caffeic acid from other sources than Pycnogenol^®^ were to be expected in the blood samples.

In contrast, the adherence to the study medication was excellent based on the pill count-back on returned medication containers. In the Pycnogenol^®^ group the average adherence was 99.4% (range 96%–100%) for all but one study participant who apparently took only 76% of the capsules. No treatment-associated adverse effects were reported except for one patient of the Pycnogenol^®^ group who experienced flatulence.

### 3.2. Method for Analysis of Polyphenols in Human Synovial Fluid Samples

To the best of our knowledge, this is the first study describing the detection and quantification of polyphenols in human synovial fluid. Since concentrations in synovial fluid samples were possibly lower than in blood and based on the fact that a previous pharmacokinetic study revealed plasma concentrations of polyphenolic compounds in the nanomolar range after intake of Pycnogenol^®^ [5], a highly sensitive method was required. In the course of method development, the main focus was the optimal detection and quantification of the metabolite M1.

Analogous to previously developed methods for analysis of Pycnogenol^®^ polyphenols in serum and blood cells [18,19], various sample preparation techniques were compared and a liquid-liquid extraction method was chosen. For the analytes of highest interests, the lower limits of quantification (LLOQs) in synovial fluid were 0.080 ng/mL for taxifolin and 0.117 ng/mL for M1 (Table 1 and Table S9). The method was slightly less sensitive for ferulic acid (LLOQ of 1.53 ng/mL), catechin (2.14 ng/mL), and caffeic acid (3.07 ng/mL).

In the supplementary materials, details about the recovery, matrix effects, and process efficiency (Tables S10 and S11), as well as the internal standard normalized matrix factor (Table S12) in pooled and individual lots of synovial fluid are documented. The method was validated based on current EMA (European Medicines Agency) and FDA (US Food and Drug Administration) guidelines and complied with the requirements for selectivity, linearity (Table S3), precision and accuracy (Tables S6 and S7), robustness (Table S6), carry-over, cross-talk, and post-preparative stability (Tables S9 and S10).

**Figure 2:**
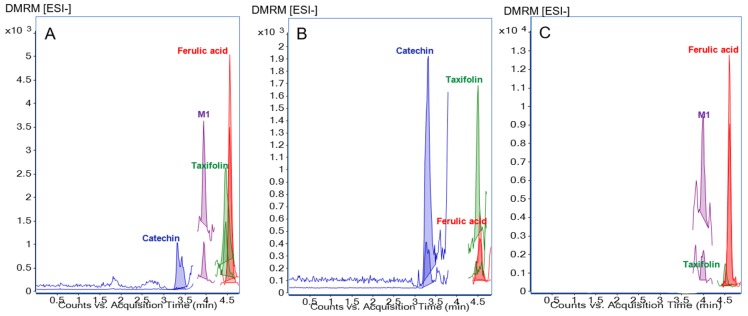
Example chromatograms for quantification in the three different sample matrices of one individual study participant after multiple dosing of 200 mg/day Pycnogenol^®^ over the course of three weeks (P+, V3). (**A**) Serum; (**B**) Blood cells; (**C**) Synovial fluid.

Representative chromatograms of all three specimens of an individual study participant after multiple dosing of 200 mg/day Pycnogenol^®^ over the course of three weeks (P+, V3) revealed total concentrations of 23.17 ng/mL catechin, 3.70 ng/mL ferulic acid, 0.19 ng/mL taxifolin, 0.16 ng/mL M1 in serum, 74.31 ng/mL catechin, 1.93 ng/mL ferulic acid, and 0.57 ng/mL taxifolin in blood cells, and 3.19 ng/mL ferulic acid, 0.18 ng/mL taxifolin, and 0.17 ng/mL M1 in synovial fluid (Figure 2).

### 3.3. Pycnogenol^®^ Constituents and Metabolites in Serum Samples

In the basal serum samples (V1) the mean total concentrations (free and conjugated) of all study participants were 27.07 ± 16.39 ng/mL catechin (mean and standard deviation), 1.80 ± 2.63 ng/mL for M1, 0.07 ng/mL (*n* = 1) for taxifolin, 6.40 ± 2.58 ng/mL for ferulic acid, and 18.58 ± 6.32 ng/mL for caffeic acid. For example, catechin was detectable in 29 out of 30 V1 samples, and in 24 samples the concentrations were above 10 ng/mL. Thereby, catechin was primarily present as glucuronide-/sulfate-conjugate. When only the free concentrations were regarded, catechin was detectable in 20 out of 30 V1 samples, and in 11 samples the concentrations were above 10 ng/mL. There were no apparent differences in the basal concentrations between the participants assigned to the P+ or Co group. Even when disregarding those patients who admitted a violation of the dietary restrictions there were still considerable basal concentrations present in serum.

The analysis of serum samples obtained after three weeks (V3) of Pycnogenol^®^ intake revealed the highest concentrations for catechin, followed by caffeic acid, ferulic acid, M1, and taxifolin (Table 2, panel A). Although there was a tendency of higher concentrations of catechin in the P+ compared to the control group, the serum concentrations of M1 and caffeic acid in the control group exceeded those in the P+ group (Table 2, panel A). When patients who admitted intake of, for example, coffee, green tea, or chocolate were excluded from the analysis the trend of higher catechin levels as well as clearly higher concentrations of M1 in the P+ group became obvious and caffeic acid was not even detectable in the control group (Table 2, panel B).

The degree of analyte conjugation with sulfate and glucuronic acid in serum was determined for all samples (V1 and V3) and ranged from 54.29% ± 26.77% for catechin (*n* = 51) to 98.34% ± 4.40% for M1 (*n* = 30; Table 3).

### 3.4. Pycnogenol^®^ Constituents and Metabolites in Blood Cell Samples

As seen with the serum samples before, basal total concentrations of the analytes with the exception of caffeic acid were detectable at V1, with no differences between the participants assigned to the P+ or Co group. Mean concentrations of 61.38 ± 40.25 ng/mL catechin (mean ± SD), 1.68 ± 0.55 ng/mL ferulic acid, 0.40 ± 0.18 ng/mL taxifolin, and 0.19 ± 0.08 ng/mL M1 were determined.

The analysis of blood cell samples obtained after three weeks (V3) of intake of Pycnogenol^®^ revealed the highest concentrations for catechin, followed by ferulic acid, M1, and taxifolin (Table 2, panel A). No caffeic acid was detectable in any of the samples. There were no clear differences in the concentrations determined in the P+ or Co group. When patients who admitted non-adherence to the dietary restrictions were excluded from the analysis there was a slight trend towards higher catechin, taxifolin, and ferulic acid levels in the P+ group compared to the control group (Table 2, panel B).

In the V3 samples of the P+ group, the open-chained ester form of M1 (M1-COOH; *n* = 5; Figure S1A) was identified as well as the glutathione conjugate of M1 (M1-GSH; *n* = 1; Figure S1B). In the V3 samples of the Co group, only the M1-COOH was detected in one patient sample.

### 3.5. Pycnogenol^®^ Constituents and Metabolites in Synovial Fluid Samples

In the present study, synovial fluid samples were obtained at the time of knee surgery. The analysis revealed the highest concentrations for caffeic acid, followed by ferulic acid, catechin, M1, and taxifolin (Table 2, panel A). With the exception of taxifolin, there were no vast differences in the concentrations of the other polyphenols.

### 3.6. Distribution of Pycnogenol^®^ Constituents and Metabolites between Specimen

**Figure 3:**
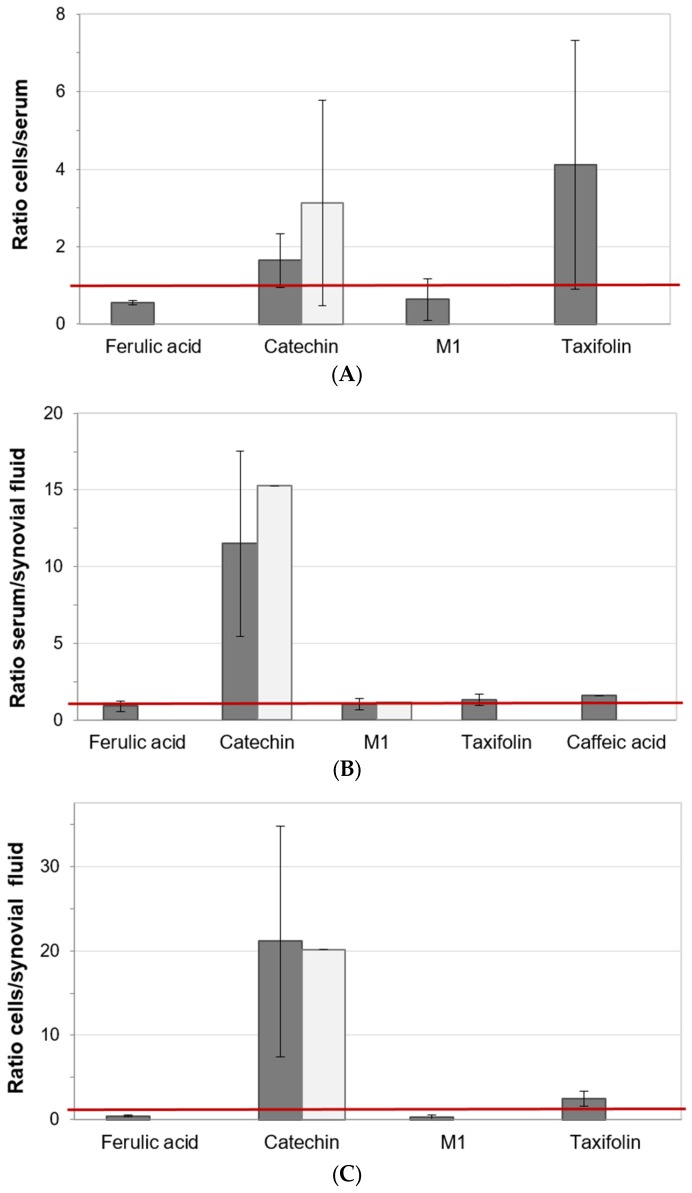
Summarized individual ratios of the analyte concentrations in different specimens of single study participants. Columns of the intervention (P+; dark gray) and control (Co; light grey) group represent the mean and standard deviation of the individual calculated ratios. (**A**) Ratio cells/serum. (**B**) Ratio serum/synovial fluid. (**C**) Ratio cells/synovial fluid.

To exclude the chance that individual trends in distribution of the analytes were overlooked if only group mean concentrations were considered, the individual ratios of the analyte concentrations in the different specimen of single patients were calculated and summarized (Figure 3).

The mean and standard deviations of the individual concentration ratios of the analytes in blood cells and serum (total concentrations, V3) showed that ferulic acid (0.56 ± 0.06; *n* = 4), M1 (0.64 ± 0.54; *n* = 5), and taxifolin (4.11 ± 3.21; *n* = 5) were detected in both matrices exclusively in the P+ group (Figure 3, panel A). With ratios above 1.0, taxifolin and catechin were clearly more present in blood cells compared to serum. In the patient group receiving 200 mg/day Pycnogenol^®^, the catechin distribution into blood cells (1.65 ± 0.69; *n* = 11) was less pronounced than in the control group (3.14 ± 2.65; *n* = 7).

The individual concentration ratios of analytes in serum and synovial fluid revealed that in both patient groups catechin was primarily distributed into serum compared to the synovial fluid (P+: 11.51 ± 6.04; *n* = 4 and Co: 15.27; *n* = 1; Figure 3, panel B). Caffeic acid (1.60; *n* = 1), taxifolin (1.33 ± 0.38; *n* = 2), and ferulic acid (0.89 ± 0.32; *n* = 4) were present in both matrices only after intake of Pycnogenol^®^ and not in the control group. Ferulic acid preferentially resided in the synovial fluid, while taxifolin and caffeic acid showed the opposite tendency. The metabolite M1 was detected both in the P+ (1.01 ± 0.37; *n* = 3) and Co group (1.14; *n* = 1) and it appeared to be almost in equilibrium between serum and synovial fluid.

The individual analyte distribution between blood cells and synovial fluid revealed a strong tendency of catechin for localization within blood cells compared to the synovial fluid (Figure 3, panel C). This was observed in both groups of the study participants (P+: 21.11 ± 13.70; *n* = 2 and Co: 20.15; *n* = 1). Taxifolin (2.48 ± 0.90; *n* = 2), M1 (0.27 ± 0.27; *n* = 2), and ferulic acid (0.45 ± 0.15; *n* = 5)) were present in both matrices exclusively in the P+ group. With a distribution ratio higher than 1.0, taxifolin was more present in blood cells compared to synovial fluid, while M1 and ferulic acid preferentially resided in the synovial fluid.

## 4. Discussion

In the present study, the in vivo distribution of constituents and metabolites of the maritime pine extract Pycnogenol^®^ between human serum, blood cells, and synovial fluid was investigated for the first time. A newly developed and validated highly sensitive LC-ESI/MS/MS method allowed for the detection and quantification of various polyphenolic compounds in synovial fluid and thereby facilitated the proof that polyphenols are actually distributed into joints.

Analysis of samples obtained before the start of the intervention (V1) revealed that catechin and other polyphenols were ubiquitously present in human serum samples at measurable basal levels. Similar observations have been reported by others [21]. Based on the fact that the most controlled condition (lifestyle/diet, timely intake of Pycnogenol^®^ capsules) the patients were subjected to was after their hospitalization (V3) when they were under observation of a study nurse, data analysis focused on V3 samples comparing the intervention (P+) with the control group (Co).

As already observed in an earlier pharmacokinetic study [5], not all polyphenols were discovered in biofluid samples of all participants, which reflected the high interindividual variability of absorption, distribution, metabolism, and/or elimination of plant constituents. The fact that the time between the last intake of Pycnogenol^®^ and the collection of the specimen slightly varied around 12 h might have also contributed to the variability of measured compound concentrations. Although the mean polyphenol concentrations in serum, blood cells, and synovial fluid were similar in the P+ and Co group, due to non-adherence to the dietary restrictions, distinctive observations were made in the P+ group.

In serum samples, taxifolin and ferulic acid were only detectable in the P+, not in the Co group. In a previous pharmacokinetic study with healthy volunteers, taxifolin was not detectable under steady state conditions, which was most probably due to the less sensitive analytical method [5]. This is consistent with the very low concentrations of taxifolin found in the present study. Ferulic acid has been suggested to be a marker of consumption of maritime pine bark extract. In healthy volunteers adhering to a low polyphenol diet, both free and conjugated ferulic acid were determined in urine samples [22].

The conjugation degree of the investigated polyphenols in serum was high and ranged from 54.29% ± 26.77% for catechin to 98.34% ± 4.40% for M1. This is consistent with the data determined in a former investigation [5]. However, as observed before and reported by others [22], the interindividual variability of the conjugation degree of the analytes was high. Even in the individual person the degree of conjugation cannot be regarded as a constant since it apparently also depends on the current analyte concentration in the specimen [23].

Blood cells and erythrocytes represent a significant pharmacological compartment for distribution of xenobiotics [24,25]. Individual polyphenols have been shown to accumulate in human blood cells, macrophage-derived foam, or endothelial cells [15,16,25,26]. Notably, only low concentrations of M1 were found in the cellular fraction of the blood samples. This apparently contradicts previous results showing an enhanced cellular uptake of M1, possibly via the GLUT-1 transporter, into human erythrocytes [16]. However, M1 is subsequently subjected to an extensive intracellular metabolism [17] which would explain the low remaining intracellular levels of M1 under steady state conditions. Consequently, the blood cells samples of the present study were also screened for the presence of any of the previously detected cellular M1 metabolites [17]. Indeed, in the P+ group, the open-chained ester form of M1 (M1-COOH) and the glutathione conjugate of M1 (M1-GSH) were identified, though not quantified. In the Co group, only M1-COOH was detected.

Sampling of synovial fluid is typically practiced for diagnostic reasons (e.g., for detection of a septic arthritis). In research, there is great interest in osteoarthritis biomarkers such as cytokines that might assist diagnosis or prognosis [27]. In contrast, drug concentrations are rarely reported for synovial fluid samples. To the best of our knowledge, this is the first study investigating polyphenol concentrations in human synovial fluid samples of patients with osteoarthritis. Similar to the results of serum analysis, mean polyphenol concentrations were similar in the P+ and Co groups, but taxifolin was only detectable in the P+ group. Thus, it might be a marker of Pycnogenol^®^ consumption.

Comparing mean concentrations of the constituents and metabolites in serum, blood cells, and synovial fluid revealed that the individual compounds did not distribute equally between the specimen. Notably, the highest concentrations of the polyphenols were not detected in serum. Catechin and taxifolin primarily resided within the blood cells, while M1, ferulic, and caffeic acid were mainly present in synovial fluid samples. Generally, data on distribution of polyphenols in humans is scarce. Although numerous investigations focus on absorption, metabolism, and elimination of polyphenols [1,28], only a few studies investigate the distribution, for example, into human tissues [29,30]. Distribution into or accumulation in certain body compartments might help with understanding the effects of polyphenols despite the typically low plasma/serum concentrations that are usually observed [1,29].

Although there were some trends towards higher analyte concentrations in the specimen of the patients who received Pycnogenol^®^ in the present study, the mean concentrations were similar for both groups and were subject to high interindividual variability. It was possible that individual trends in distribution of the analytes could have been overlooked if only group mean concentrations were considered. Therefore, the individual ratios of the analyte concentrations in the different specimens of single patients were calculated and summarized. Since ratios higher than 1 indicate that the analyte is primarily distributed into, for example, blood cells compared to serum, it can be concluded that higher concentrations of taxifolin and catechin were present in blood cells while ferulic acid and M1 preferentially resided in serum. This observation confirms the differential distribution of polyphenolic compounds between serum, blood cells, and synovial fluid that was already seen when mean concentrations were examined. On an individual level, a simultaneous presence of ferulic acid, M1, and taxifolin in blood cells and serum or cells and synovial fluid was only observed after intake of the pine bark extract. Also, ferulic acid, taxifolin, and caffeic acid were only detected in both serum and synovial fluid in the P+ group.

The fact that the control group received no placebo capsules to conceal the allocation to the intervention group could be seen as a potential study limitation. However, the primary interest was the bioanalysis of polyphenols in different specimens, and it is highly unlikely that any of the analyte concentrations were deliberately influenced by the patients’ knowledge of group allocation. All analytical procedures were fully validated and left no room for subjective data interpretation. A study limitation was the small group size. Since not all polyphenols were present in the biofluid samples of all patients due to interindividual pharmacokinetic differences and also as a result of the non-adherence of the participants to the dietary restrictions, a statistical differentiation between the groups was not feasible. However, the results of the present study provide a basis for sample size calculations in future studies. Those studies would then be sufficiently powered for statistical analysis and could reliably uncover significant differences between treatment and control groups. The fact that the study participants of the present study did not follow the dietary suggestions improves the generalizability of the results since they mirror real life conditions under which foods or beverages rich in polyphenols are occasionally or regularly consumed.

## 5. Conclusion

The results of this study provide the first evidence that polyphenols distribute into the synovial fluid of patients with osteoarthritis, which supports rationalizing the results of clinical efficacy studies.

## Figures and Tables

**Table 1:** Lower limits of quantification (LLOQs) of polyphenolic analytes of highest interest. Data for serum and blood cells was derived from previous work [18,19].

Analyte	LLOQ Synovial Fluid (ng/mL)	LLOQ Serum (ng/mL)	LLOQ Blood Cells (ng/mL)
Catechin	2.14	5.86	28.90
M1	0.12	0.16	0.12
Taxifolin	0.08	0.06	0.12
Caffeic Acid	3.07	8.22	48.40
Ferulic Acid	1.53	2.74	0.97

**Table:** 

A		Catechin		M1		Taxifolin		Ferulic Acid		Caffeic Acid	
		Conc. (ng/mL) ± SD		Conc. (ng/mL) ± SD		Conc. (ng/mL) ± SD		Conc. (ng/mL) ± SD		Conc. (ng/mL) ± SD	
Serum	P+	52.53 ± 18.40	*n* = 15	0.54 ± 0.84	*n* = 9	0.20 ± 0.12	*n* = 5	3.02 ± 0.39	*n* = 7	9.28 ± 0.51	*n* = 3
	Co	45.85 ± 39.59	*n* = 15	1.07 ± 1.09	*n* = 5	n.d.	-	n.d.	-	14.84 ± 1.92	*n* = 3
Blood Cells	P+	71.18 ± 27.34	*n* = 14	0.19 ± 0.07	*n* = 12	0.52 ± 0.23	*n* = 15	1.86 ± 0.36	*n* = 10	n.d.	-
	Co	70.48 ± 36.22	*n* = 13	0.21 ± 0.05	*n* = 5	0.48 ± 0.32	*n* = 15	1.80 ± 0.85	*n* = 7	n.d.	-
Synovial Fluid	P+	2.99 ± 0.43	*n* = 4	0.62 ± 0.77	*n* = 5	0.21 ± 0.03	*n* = 2	4.29 ± 1.83	*n* = 8	10.32 ± 3.96	*n* = 7
	Co	3.94 ± 1.83	*n* = 2	0.78 ± 0.74	*n* = 4	n.d.	-	3.04 ± 0.79	*n* = 6	12.83 ± 8.95	*n* = 10

**Table:** 

B		Catechin		M1		Taxifolin		Ferulic Acid		Caffeic Acid	
		Conc. (ng/mL) ± SD		Conc. (ng/mL) ± SD		Conc. (ng/mL) ± SD		Conc. (ng/mL) ± SD		Conc. (ng/mL) ± SD	
Serum	P+	48.41 ± 18.61	*n *= 11	0.70 ± 1.02	*n *= 6	0.20 ± 0.12	*n *= 5	3.09 ± 0.46	*n *= 5	9.78	*n *= 1
	Co	32.24 ± 17.38	*n *= 8	0.25 ± 0.05	*n *= 2	n.d.	-	n.d.		n.d.	*-*
Blood Cells	P+	73.75 ± 29.25	*n *= 11	0.20 ± 0.07	*n *= 9	0.56 ± 0.19	*n *= 11	1.85 ± 0.38	*n *= 9	n.d.	-
	Co	63.31 ± 31.28	*n *= 7	0.18 ± 0.05	*n *= 2	0.39 ± 0.16	*n *= 8	1.69 ± 0.10	*n *= 3	n.d.	-
Synovial Fluid	P+	3.00 ± 0.58	*n *= 2	0.92 ± 0.93	*n *= 3	0.21 ± 0.03	*n *= 2	4.31 ± 2.10	*n *= 6	10.63 ± 3.86	*n *= 4
	Co	2.65	*n *= 1	0.17 ± 0.03	*n *= 2	n.d.	-	3.16 ± 0.22	*n *= 3	10.99 ± 5.79	*n *= 4

**Table 3:** Mean and standard deviation (SD) of the conjugation degree in serum samples (both P+ and Co group; V1, V2, and V3 blood samples; in total *n* = 90 samples). Results were compared with former investigations [5].

Analytes	Conjugation Degree (%)					
	Current Study			Former Investigation		
	Mean	± SD	Sample Size	Mean	± SD	Sample Size
Catechin	54.29	26.77	*n* = 51	56.50	27.90	*n* = 5
M1	98.34	4.40	*n* = 30	100 *		
Taxifolin	96.75	7.23	*n* = 11	100 *		
Ferulic Acid	90.32	16.58	*n* = 24	100 *		
Caffeic Acid	80.95	17.95	*n* = 10	69.40	11.80	*n* = 3

* A conjugation degree of 100% was assumed because no free concentrations were detectable.

## Supplementary Materials

The following are available online at http://www.mdpi.com/2072-6643/9/5/443/s1, Figure S1: Example chromatograms for identification of intracellular metabolites of M1 in blood cells of study participants after multiple dosing of 200 mg/day Pycnogenol^®^ over the course of three weeks (P+, V3), Table S1: Additional optimized transitions and parameters in dynamic multiple reaction monitoring (DMRM) mode for identification of compounds in human blood cells, Table S2: Optimized transitions and parameters in dynamic multiple reaction monitoring (DMRM) employing negative ESI ionization mode for LC-MS/MS analysis of prepared synovial fluid samples, Table S3: Calibration range, calibration function and correlation coefficients of the five analytes extracted from human pooled synovial fluid, Table S4: Intraday accuracy and precision of the analytes in human pooled synovial fluid, Table S5: Interday accuracy and precision of the analytes in human pooled synovial fluid, Table S6: Robustness of the developed method at two concentrations with human pooled synovial fluid which was intentionally contaminated with 1% human whole blood, Table S7: Post-preparative stability: autosampler stability of the analytes after 6 h and 12 h at room temperature after previous LC/MS/MS analysis, Table S8: Post-preparative stability: stability of the analytes after one freeze-thaw cycle, Table S9: Lower limit of quantification (LLOQ) and related accuracy of the five analytes extracted from human pooled synovial fluid, Table S10: Recovery, matrix effects and process efficiency of the five analytes extracted from human pooled synovial fluid at three concentrations, Table S11: Recovery, matrix effects and process efficiency of the five analytes extracted from three lots of synovial fluid at two concentrations, Table S12: Internal standard (hydrocaffeic acid) normalized matrix factor at human pooled synovial fluid in three concentrations and in three lots of synovial fluid at two concentrations.
