# Supplementary material for: Distribution of Constituents and Metabolites of Maritime Pine Bark Extract (Pycnogenol®) into Serum, Blood Cells, and Synovial Fluid of Patients with Severe Osteoarthritis: A Randomized Controlled Trial
Source: Nutrients. 2017 Apr 28;9(5):443. doi: 10.3390/nu9050443 (PMC5452173; doi:10.3390/nu9050443)

Table S1: Additional optimized transitions and parameters in dynamic multiple reaction monitoring (DMRM) mode for identification of compounds in human blood cells. Electron multiplier voltage (EMV) was set to +500 V in ESI negative and +1000 V in ESI positive mode. Following intracellular metabolites of M1 were monitored: Conjugation products of M1 and glutathione (M1-GSH), cysteine (M1-CYS) and oxidized glutathione (M1-GSSG); an acetylated form of M1 (M1-acetylated), the open-chained ester form of M1 (M1-COOH) and another metabolite, respectively hydroxybenzoic acid.

Compound	Precursor ion *(m/z)*	Product ion *(m/z)*	FV[a] [V]	CE[b] [V]	CAV[c] [V]	MS 1 Resolution	MS 2 Resolution	R_T[d] [min]	R_T Window *(DMRM)*	ESI mode
M1-GSH	514.0	514.0	95	0	1	Widest	Widest	3.50	0.50	positive
	514.0	385.0	95	13	1	Widest	Widest	3.50	0.50	positive
	514.0	130.0	95	23	1	Widest	Widest	3.50	0.50	positive
M1-CYS	328.0	328.0	100	0	1	Widest	Widest	3.60	0.50	positive
	328.0	155.0	70	15	7	Widest	Widest	3.60	0.50	positive
	328.0	85.0	100	25	7	Widest	Widest	3.60	0.50	positive
M1-GSSG	410.0	410.0	150	0	3	Widest	Widest	3.10	0.40	positive
	410.0	232.0	135	15	7	Widest	Widest	3.10	0.40	positive
M1-acetylated	251.0	251.0	100	0	7	Widest	Widest	5.40	0.50	positive
M1-COOH	225.0	123.0	100	10	3	Widest	Widest	3.60	0.40	negative
	225.0	101.0	100	5	3	Widest	Widest	3.60	0.40	negative

[a]FV. Fragmentor voltage. [b]CE. Collision energy. [c]CAV. Cell accelerator voltage. [d]R_T. Retention time.

Table S2: Optimized transitions and parameters in dynamic multiple reaction monitoring (DMRM) employing negative ESI ionization mode for LC-MS/MS analysis of prepared synovial fluid samples. Electron multiplier voltage (EMV) was set to +750 V. Cycle time was 1000 ms.

Compound	Precursor ion *(m/z)*	Product ion *(m/z)*	FV[a] [V]	CE[b] [V]	CAV[c] [V]	MS 1 Resolution	MS 2 Resolution	R_T[d] [min]	R_T Window *(DMRM)*
(+)-Catechin	289.1	245.0[e]	76	9	7	Widest	Widest	3.60	0.45
	289.1	203.0	76	17	7	Widest	Widest	3.60	0.45
M1	207.0	163.1[e]	115	13	5	Widest	Widest	4.00	0.45
	207.0	122.0	115	17	5	Widest	Widest	4.00	0.45
Caffeic acid	179.0	135.1[e]	90	13	5	Widest	Widest	4.00	0.50
	179.0	134.0	90	25	5	Widest	Widest	4.00	0.50
Taxifolin	303.1	285.0[e]	95	9	4	Widest	Widest	4.55	0.45
	303.1	125.0	95	21	4	Widest	Widest	4.55	0.45
Ferulic acid	193.1	134.1[e]	80	13	1	Widest	Widest	4.65	0.45
	193.1	178.0	80	9	1	Widest	Widest	4.65	0.45
Hydrocaffeic acid (IS)	181.2	137.0[e]	85	9	4	Wide	Widest	3.85	0.50
	181.2	109.0	85	9	4	Wide	Widest	3.85	0.50

[a]FV. Fragmentor voltage. [b]CE. Collision energy. [c]CAV. Cell accelerator voltage. [d]R_T. Retention time. [e]Quantifier. transition for quantification

Table S3: Calibration range, calibration function and correlation coefficients of the five analytes extracted from human pooled synovial fluid (n= 3).

Analytes	Range [ng/mL]	Slope ± SD	y-intercept	Correlation coefficient R
(+)-Catechin	2.14 - 34.93	0.0195 ± 0.006	-0.0175	0.9977
Ferulic acid	1.53 - 24.93	0.1184 ± 0.024	0.0693	0.9979
M1	0.117 - 1.900	1.6016 ± 0.243	0.0330	0.9976
Taxifolin	0.080 - 1.307	0.7518 ± 0.010	-0.0388	0.9992
Caffeic acid	3.07 - 49.95	0.6752 ± 0.113	-0.8800	0.9988

Table S4: Intraday accuracy and precision of the analytes in human pooled synovial fluid (n= 3).

Analytes and spiked concentration [ng/mL]	Calculated concentration Mean ± SD [ng/mL]	Accuracy Mean ± SD [%]	Precision [%]
Catechin			
2.14	2.24 ± 0.15	104.55 ± 6.78	6.48
2.68	2.63 ± 0.43	98.24 ± 16.24	16.50
8.18	7.47 ± 0.79	91.40 ± 9.67	10.58
12.77	10.86 ± 0.41	85.04 ± 3.21	3.77
19.96	17.62 ± 0.71	88.25 ± 3.54	4.01
Ferulic acid			
1.53	1.70 ± 0.05	111.13 ± 3.45	3.10
1.91	1.98 ± 0.14	103.36 ± 7.18	6.95
5.83	5.13 ± 0.19	87.85 ± 3.28	3.73
9.12	8.60 ± 0.73	94.33 ± 7.97	8.45
14.24	13.27 ± 0.95	93.14 ± 6.68	7.17
M1			
0.117	0.129 ± 0.016	110.51 ± 13.43	12.16
0.146	0.152 ± 0.014	103.84 ± 9.37	9.02
0.445	0.457 ± 0.049	102.64 ± 10.97	10.69
0.695	0.770 ± 0.030	110.82 ± 4.25	3.84
1.086	1.014 ± 0.080	93.39 ± 7.37	7.89
Taxifolin			
0.080	0.084 ± 0.016	105.70 ± 19.48	18.43
0.100	0.096 ± 0.015	95.91 ± 15.48	16.14
0.306	0.270 ± 0.016	88.35 ± 5.11	5.78
0.478	0.427 ± 0.020	89.36 ± 4.27	4.77
0.747	0.653 ± 0.020	87.40 ± 2.72	3.12
Caffeic acid			
3.07	3.55 ± 0.18	115.73 ± 5.74	4.96
3.83	3.64 ± 0.13	95.11 ± 3.47	3.65
11.69	10.42 ± 0.70	89.15 ± 5.97	6.70
18.27	18.65 ± 1.99	102.10 ± 10.91	10.69
28.54	27.85 ± 2.17	97.57 ± 7.59	7.78

Table S5: Interday accuracy and precision of the analytes in human pooled synovial fluid (n= 3).

Analytes and spiked concentration [ng/mL]	Calculated concentration Mean ± SD [ng/mL]	Accuracy Mean ± SD [%]	Precision [%]
Catechin			
2.14	2.16 ± 0.33	100.72 ± 15.43	15.32
2.68	2.77 ± 0.16	103.53 ± 5.85	5.65
8.18	7.97 ± 0.53	97.51 ± 6.51	6.68
12.77	11.28 ± 0.40	88.34 ± 3.15	3.56
19.96	17.96 ± 0.39	89.98 ± 1.94	2.16
Ferulic acid			
1.53	1.51 ± 0.16	98.91 ± 10.71	10.82
1.91	1.88 ± 0.09	98.43 ± 4.63	4.71
5.83	5.60 ± 0.43	95.97 ± 7.31	7.62
9.12	8.76 ± 0.18	96.08 ± 2.02	2.11
14.24	13.04 ± 0.39	91.53 ± 2.71	2.97
M1			
0.117	0.119 ± 0.009	101.47 ± 7.86	7.75
0.146	0.143 ± 0.008	97.62 ± 5.59	5.73
0.445	0.446 ± 0.010	100.11 ± 2.32	2.32
0.695	0.682 ± 0.077	98.17 ± 11.03	11.23
1.086	0.975 ± 0.035	89.80 ± 3.21	3.58
Taxifolin			
0.080	0.080 ± 0.004	100.56 ± 4.92	4.89
0.100	0.096 ± 0.006	96.23 ± 6.29	6.53
0.306	0.286 ± 0.015	93.50 ± 4.93	5.28
0.478	0.441 ± 0.013	92.29 ± 2.64	2.86
0.747	0.675 ± 0.020	90.31 ± 2.66	2.94
Caffeic acid			
3.07	3.06 ± 0.42	99.90 ± 13.81	13.82
3.83	3.67 ± 0.16	95.89 ± 4.06	4.23
11.69	11.23 ± 0.86	96.08 ± 7.36	7.66
18.27	17.39 ± 1.30	95.19 ± 7.11	7.46
28.54	24.66 ± 4.35	93.19 ± 4.11	4.41

Table S6: Robustness of the developed method at two concentrations (n= 3) with human pooled synovial fluid which was intentionally contaminated with 1% human whole blood.

**Analytes and spiked concentration [ng/mL]**	**Calculated concentration Mean ± SD [ng/mL]**	**Accuracy Mean [%]**	**Precision [%]**
Catechin			
8.18	8.68 ± 0.55	106.18	6.34
19.96	20.99 ± 1.98	105.16	9.42
M1			
0.445	0.478 ± 0.02	107.37	4.55
1.086	1.033 ± 0.08	95.12	8.17
Caffeic acid			
11.69	11.30 ± 1.40	96.65	12.37
28.54	24.69 ± 0.82	86.50	3.33
Taxifolin			
0.306	0.314 ± 0.04	102.77	13.50
0.747	0.776 ± 0.11	103.88	14.06
Ferulic acid			
5.83	6.50 ± 0.12	111.40	1.91
12.24	15.70 ± 0.49	110.21	3.11

Table S7: Post-preparative stability: autosampler stability of the analytes after 6 h and 12 h at room temperature (RT) after previous LC/MS/MS analysis (n= 3).

Analytes and spiked concentration [ng/mL]	Autosampler stability: 6 h - RT - in darkness			Autosampler stability: 12 h - RT - in darkness		
	Calculated concentration Mean ± SD [ng/mL]	RSD [%]	Δ [%][1] Mean ± SD	Calculated concentration Mean ± SD [ng/mL]	RSD [%]	Δ [%][1] Mean ± SD
(+)-Catechin						
2.68	2.72 ± 0.34	12.54	1.35	2.78 ± 0.34	12.32	3.71
8.18	7.84 ± 1.20	15.28	-4.16	7.21 ± 0.72	9.96	-11.87
12.77	10.72 ± 1.51	14.04	-16.04	10.92 ± 1.50	13.72	-14.50
19.96	17.71 ± 1.11	6.27	-11.25	16.10 ± 0.83	5.17	-19.36
M1						
0.146	0.151 ± 0.02	14.28	3.09	0.157 ± 0.01	7.21	7.24
0.445	0.457 ± 0.04	9.07	2.66	0.437 ± 0.01	3.06	-1.90
0.695	0.700 ± 0.08	11.06	0.73	0.749 ± 0.07	9.70	7.72
1.086	1.172 ± 0.06	4.70	7.96	1.097 ± 0.10	8.71	1.04
Caffeic acid						
3.83	3.73 ± 0.02	0.47	-2.76	3.71 ± 0.25	6.77	-3.14
11.69	11.26 ± 1.71	15.18	-3.66	10.15 ± 0.84	8.25	-13.17
18.27	15.86 ± 0.08	5.07	-13.16	15.71 ± 0.59	3.74	-13.99
28.54	26.80 ± 2.60	9.69	-6.10	25.24 ± 2.30	9.12	-11.58
Taxifolin						
0.100	0.107 ± 0.01	13.99	6.75	0.104 ± 0.01	12.53	4.06
0.306	0.268 ± 0.03	11.48	-12.57	0.277 ± 0.03	9.41	-9.46
0.478	0.398 ± 0.08	2.10	-16.70	0.430 ± 0.06	15.09	-10.06
0.747	0.744 ± 0.04	5.01	-0.46	0.666 ± 0.04	6.59	-10.80
Ferulic acid						
1.91	1.87 ± 0.29	15.63	-2.19	1.71 ± 0.19	10.86	-10.61
5.83	5.57 ± 0.79	14.20	-4.49	4.95 ± 0.25	5.02	-15.15
9.11	9.01 ± 0.51	5.68	-1.15	8.49 ± 0.83	9.80	-6.82
14.24	13.50 ± 0.83	6.12	-5.18	12.91 ± 0.26	2.00	-9.37

[1]: (calculated concentration mean ± SD [ng/mL] / (spiked concentration [ng/mL])-1)*100

Table S8: Post-preparative stability: stability of the analytes after one freeze-thaw cycle (n= 3).

Analytes and spiked concentration [ng/mL]	Freeze-thaw stability: 1 cycle -20 °C h/at least 12 h - RT/1h		
	Calculated concentration Mean ± SD [ng/mL]	RSD [%]	Δ [%][1] Mean ± SD
(+)-Catechin			
2.68	2.09 ± 0.16	7.81	-21.82
8.18	7.42 ± 0.59	7.52	-9.29
12.77	10.63 ± 0.34	3.21	-16.76
19.96	17.78 ± 2.66	14.96	-10.93
M1			
0.146	0.209 ± 0.00	0.99	42.87
0.445	0.564 ± 0.05	8.22	26.63
0.695	0.737 ± 0.06	8.64	6.02
1.086	1.180 ± 0.176	14.95	8.66
Caffeic acid			
3.83	4.04 ± 0.37	9.18	5.58
11.69	9.92 ± 0.43	4.38	-15.13
18.27	15.50 ± 0.39	2.51	-15.14
28.54	23.55 ± 1.85	7.87	-17.50
Taxifolin			
0.100	0.107 ± 0.01	5.56	7.45
0.306	0.352 ± 0.02	6.12	14.98
0.478	0.466 ± 0.07	14.48	-2.48
0.747	0.742 ± 0.12	15.95	-0.70
Ferulic acid			
1.91	2.24 ± 0.12	5.51	16.91
5.83	6.11 ± 0.59	9.64	4.80
9.11	9.94 ± 0.95	9.55	9.04
14.24	13.29 ± 1.89	14.20	-6.72

[1]: (calculated concentration mean ± SD [ng/mL] / (spiked concentration [ng/mL])-1)*100

Table S9: Lower limit of quantification (LLOQ) and related accuracy of the five analytes extracted from human pooled synovial fluid (n= 3).

**Analytes**	**LLOQ [ng/mL]**	**Accuracy $_{LLOQ}$ [%] Mean ± SD**
(+)-Catechin	2.14	101.41 ± 17.00
Ferulic acid	1.53	93.39 ± 14.38
M1	0.117	97.24 ± 17.38
Taxifolin	0.080	103.15 ± 12.49
Caffeic acid	3.07	106.85 ± 8.31

Table S10: Recovery, matrix effects and process efficiency of the five analytes extracted from human pooled synovial fluid at three concentrations (n= 3).

Analytes and spiked concentration [ng/mL]	Recovery [%]			Process efficiency [%]			Matrix effects [%]		
	Mean	± SD	RSD [%]	Mean	± SD	RSD [%]	Mean	± SD	RSD [%]
Catechin									
8.18	39.24	3.04	7.75	29.03	2.09	7.21	-26.00	0.71	2.72
12.77	45.16	8.01	17.74	33.91	4.75	14.02	-24.91	2.91	11.70
19.96	40.81	2.41	5.90	32.37	2.62	8.10	-20.68	3.11	15.05
Taxifolin									
0.306	63.24	6.12	9.67	95.25	7.56	7.94	50.62	1.40	2.76
0.478	87.63	7.10	8.12	122.00	11.85	9.72	39.23	1.78	4.53
0.747	71.76	7.24	10.08	109.86	10.86	9.88	53.10	3.21	6.05
M1									
0.445	45.76	4.16	9.10	165.99	11.92	7.18	262.77	15.11	5.75
0.695	49.17	9.72	19.78	172.14	14.08	8.18	250.13	27.75	11.09
1.086	55.43	3.23	5.82	192.04	11.09	5.78	246.45	5.67	2.30
Ferulic acid									
5.83	52.47	7.32	13.94	149.25	19.75	13.23	184.42	2.45	1.33
9.11	58.14	8.00	13.75	155.37	17.05	10.97	167.22	5.40	3.23
14.24	57.54	1.35	2.34	148.15	3.44	2.32	157.48	0.42	0.27
Caffeic acid									
11.69	59.92	1.59	2.66	76.51	4.20	5.50	27.69	3.90	14.07
18.27	68.30	11.71	17.15	88.63	13.96	15.75	29.77	1.94	6.50
28.54	67.48	7.65	11.33	84.63	9.67	11.43	25.41	2.84	11.16

Table S11: Recovery, matrix effects and process efficiency of the five analytes extracted from three lots of synovial fluid at two concentrations (n= 3).

Analytes and spiked concentration [ng/mL]	Recovery [%]			Process efficiency [%]			Matrix effect [%]		
	Mean	± SD	RSD [%]	Mean	± SD	RSD [%]	Mean	± SD	RSD [%]
Catechin									
8.18	43.46	6.41	14.74	31.23	3.49	11.19	-27.79	4.97	17.87
19.96	48.17	3.24	6.72	36.92	1.97	5.34	-23.30	2.21	9.47
Taxifolin									
0.306	58.16	13.15	22.61	87.37	17.02	19.48	51.13	8.12	15.87
0.747	69.25	2.62	3.78	110.24	7.98	7.24	59.05	5.97	10.11
M1									
0.445	47.45	11.15	23.51	164.55	47.69	28.98	243.83	21.48	8.81
1.086	59.38	0.68	1.14	200.23	17.52	8.75	237.04	26.50	11.18
Ferulic acid									
5.83	46.82	7.58	16.19	130.57	17.40	13.33	180.61	27.99	15.49
14.24	48.47	3.45	7.13	128.48	9.16	7.13	165.07	3.01	1.82
Caffeic acid									
11.69	63.83	8.23	12.89	80.69	11.38	14.10	26.30	1.49	5.65
28.54	62.98	6.85	10.88	81.68	11.75	14.38	29.36	5.28	18.00

Table S12: Internal standard (IS; hydrocaffeic acid) normalised matrix factor (MF) at human pooled synovial fluid in three concentrations (n= 3) and in three lots of synovial fluid at two concentrations (n= 3).

Analytes and spiked concentration [ng/mL]	IS-normalised MF in human pooled synovial fluid			IS-normalised MF in three lots of human synovial fluid		
	Mean	± SD	RSD [%]	Mean	± SD	RSD [%]
Catechin						
8.18	-0.236	0.01	3.08	-0.252	0.05	21.05
12.77	-0.229	0.04	18.11			
19.96	-0.195	0.03	16.68	-0.215	0.03	14.46
Taxifolin						
0.306	0.459	0.02	3.48	0.459	0.05	10.92
0.478	0.360	0.04	11.09			
0.747	0.501	0.05	9.66	0.540	0.03	6.18
M1						
0.45	2.380	0.21	8.61	2.207	0.28	12.74
0.70	2.297	0.28	12.29			
1.09	2.326	0.14	6.17	2.174	0.26	11.84
Ferulic acid						
5.830	1.671	0.08	4.97	1.638	0.32	19.45
9.110	1.536	0.09	5.82			
14.240	1.486	0.06	3.95	1.514	0.05	3.56
Caffeic acid						
11.69	0.251	0.03	11.11	0.237	0.00	1.41
18.27	0.273	0.01	2.60			
28.54	0.240	0.03	13.93	0.269	0.05	17.20

Figure S1: Example chromatograms for identification of intracellular metabolites of M1 in blood cells of study participants after multiple dosing of 200 mg/day Pycnogenol® over the course of three weeks (P+, V3). After initially smoothing (Gaussian, 5 points) the signal-to-noise ratio (SNR; peak-to-peak height) was calculated.

A. M1-COOH. The SNR was 47 based on calculation of the transition 223 > 123 (ESI negative).

B. M1-GSH. The SNR was 5 based on calculation of the transition 514 > 385 (ESI positive).

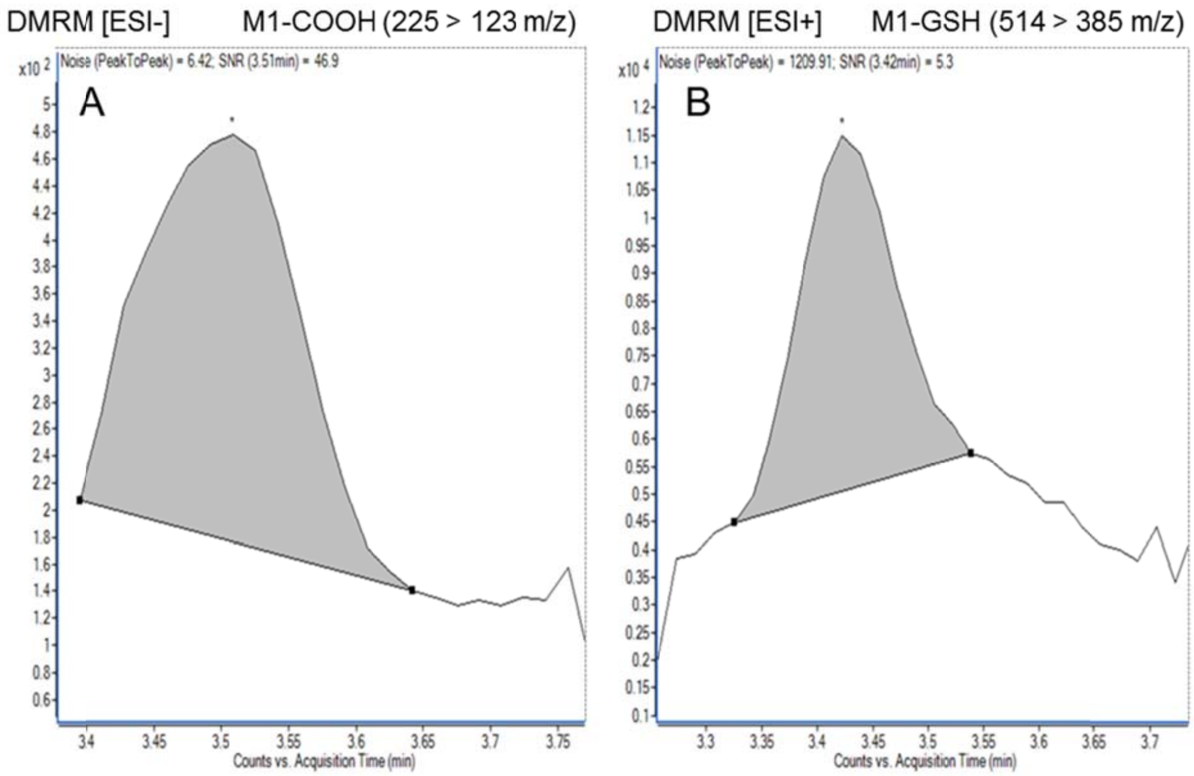

Supplement: Supplementary file 1 [file nutrients-09-00443-s001.pdf]
